# Designing care at home as an integrated continuum: insights from Singapore and application to U.S. health systems

**DOI:** 10.1038/s44401-026-00153-5

**Published:** 2026-10-07

**Authors:** Snehal A. Patel, Ron C. Li

**Affiliations:** 1 Saena Partners, Singapore, Singapore; 2 Division of Hospital Medicine, Department of Medicine, Stanford University School of Medicine, Stanford, CA USA

**Keywords:** Health care, Health humanities

## Abstract

Many U.S. hospital-at-home programs operate primarily as substitutes for inpatient admission. Singapore has instead pursued a nationally coordinated care-at-home framework: Healthier SG re-anchors chronic disease management in primary care, while MIC@Home mainstreams acute hospital care at home. We examine the governance, financing, workforce, and digital infrastructure designed to connect these programs, and draw design insights for U.S. health systems building integrated care-at-home continuums.

## Introduction

Health systems across high-income countries confront a structural mismatch between the dominant burden of disease and the organization of care delivery. Chronic conditions such as diabetes, cardiovascular disease, and hypertension account for most of the morbidity and health-care spending, yet care models remain oriented around hospitals designed for episodic, acute interventions^1^. Patients with chronic illness frequently cycle through hospitals during acute flare-ups, while long stretches of disease management occur with limited continuity of care and fragmented follow-up^2^.

In the United States, hospital at home (HaH) programs have gained traction as a mechanism to deliver inpatient-level care in patients’ homes. Adoption accelerated during the COVID-19 pandemic under the Centers for Medicare & Medicaid Services (CMS) Acute Hospital Care at Home waiver, and hundreds of hospitals operate such programs^3,4^. Yet many U.S. HaH programs still function primarily as point solutions: they substitute for a hospital bed without connecting to upstream chronic disease management or downstream recovery and prevention, which are already conducted primarily outside of the hospital. The result is improved efficiency and hospital capacity creation at the margins, but without fully leveraging these programs as part of an integrated care continuum to facilitate safe and effective transition between acute and chronic care.

Singapore, while also investing in individual hospital at home programs, has followed a broader national vision to move care to the home, with the stated aims of slowing chronic disease progression, reducing avoidable acute hospital utilization, and containing long-term cost growth. Two initiatives are central to this effort. Healthier SG, launched nationally in 2023, is a population-level strategy to re-anchor chronic disease management in primary care through enrollment with a regular family physician, personalized health plans, and community-based preventive programs^5^. Mobile Inpatient Care at Home (MIC@Home), first piloted in 2019 and mainstreamed across Singapore’s public acute hospital system from April 1, 2024, delivers hospital-level acute care in patients’ homes as a substitute for selected inpatient admissions^6,7^. Taken together, these reforms reflect a nationally coordinated approach to care at home that spans inpatient, ambulatory, and population health management strategies, in contrast to the United States, where care at home has largely grown out of individual health systems’ hospital at home programs responding to inpatient bed shortages. This article examines the design of Singapore’s intended model and draws insights for U.S. health systems developing strategies for care at home that may extend beyond only HaH programs that cover the acute care episode.

## Singapore’s Integrated Approach to Community-Based Care

Singapore’s approach is organized around a national policy framework intended to run from population-level prevention, through chronic disease management in primary care, to acute hospital care delivered in patients’ homes. Three structural choices underpin this design. First, a 2017 reorganization vested Singapore’s three regional health clusters with end-to-end accountability for primary, community, acute, and post-acute care within their geographies. Second, an MOH innovation office (MOHT, established 2018) designs and scales cross-setting care models, including both the Future Primary Care program that underpins Healthier SG and the Integrated General Hospital program that houses MIC@Home. Third, a set of shared protocols, redesigned workforce roles, and a national digital backbone connects community providers to hospitals. Healthier SG and MIC@Home are the most visible expressions of this continuum at its upstream and downstream poles, not standalone programs.

### A. Policy context: the “Three Beyonds”

Singapore’s health system has long emphasized efficiency and cost containment, but demographic aging and rising chronic disease prevalence have exposed the limits of the city-state’s hospital-centric orientation. In 2016, MOH introduced the “Three Beyonds”: moving beyond healthcare to health, beyond hospital to community, and beyond quality to value—a strategic reframing that catalyzed structural reorganization^8^.

In January 2017, MOH consolidated six regional health groupings into three integrated regional clusters: SingHealth (East), National Healthcare Group/NHG (Central), and National University Health System/NUHS (West). Each cluster was given full vertical accountability spanning primary care, community hospitals, and acute general hospitals within a defined geography of approximately 1.5 million residents^9,10^. These clusters now also serve as Regional Health Managers (RHMs), and they are the operational substrate for both Healthier SG, which they administer in partnership with private GPs, and MIC@Home, which they deliver through their tertiary acute hospitals under brand variants such as SGH@Home, NUHS@Home, TTSH@Home, and CGH@Home. The MOH Office for Healthcare Transformation (MOHT) was established the following year (2018) to design system-level care models that span hospital and community settings^11,12^. These national initiatives provided the policy and structural context within which both Healthier SG and MIC@Home were developed as components of a transformation away from hospital-centric care.

### B. Healthier SG: re-anchoring chronic disease management in primary care

Healthier SG, a national population health strategy launched in 2023, serves as a primary care anchor of Singapore’s “Three Beyonds” integrated care at home framework as it relates to upstream chronic disease management in the community. The initiative builds on a decade of antecedent reforms, including the Chronic Disease Management Programme (introduced from 2006), the Community Health Assist Scheme (CHAS), and the Primary Care Networks (PCNs, piloted from 2012 and scaled from 2018), which together created a substrate of public-private partnership in which MOH-funded nurses and care coordinators support private GP clinics in delivering team-based chronic disease management^13^. Healthier SG layered onto this substrate a national enrollment mechanism, capitated payment to family doctors, shared care protocols, and population-based accountability—encouraging residents aged 40 and above to enroll with a regular family physician, co-develop personalized health plans, and participate in community-based preventive programs^14^.

Enrollment is voluntary but strongly incentivized. Residents who enroll receive subsidized preventive services, enhanced coverage for chronic disease management, and access to chronic medications at rates comparable to public polyclinics. Patients can use national medical savings accounts (MediSave) to cover outpatient chronic care costs^15,16^. From the state’s perspective, enrollment creates a defined population for which primary care providers assume longitudinal responsibility, enabling population-based planning and accountability.

A central pillar of Healthier SG is payment reform. Historically, primary care in Singapore relied heavily on fee-for-service billing. Under Healthier SG, participating family doctors in private practice receive an annual service fee for each enrolled resident, with payments tiered by factors including the resident’s risk profile, the scope of care required, and progress in preventive care or chronic disease management^5,14^. This population-based funding model aims to align provider incentives with long-term health outcomes rather than visit and prescription dispensing volume. Care protocols have been developed for common chronic conditions (including diabetes mellitus, hypertension, and lipid disorders) to promote greater consistency across providers^17^.

As of May 2026, more than 1.4 million residents (59% of the eligible population aged 40 and above) had enrolled^18^. Enrollment, however, is an uptake statistic, not a measure of engagement or outcome, and further studies are needed to evaluate impact on outcomes. Early engagement data are mixed: 83% of enrollees have completed a first Health Plan, but only 66% of those due for an annual check-in in 2025 completed one, and MOH has introduced financial incentives to encourage regular check-ins^18^. Early quasi-experimental evidence suggests enrollment increases preventive screening uptake^19^. Visit adherence over time, medication continuity, and intermediate clinical measures such as blood pressure or glycemic control remain unmeasured in published data. Implementation has surfaced tensions to be worked through: providers report increased administrative burden, and the long-term fiscal implications of expanded subsidies remain an open policy question^20^.

### C. MIC@Home: Acute Hospital Care in the Community

While Healthier SG addresses the chronic disease management and prevention end of the care continuum, MIC@Home addresses the acute care end, and is the flagship implementation of MOHT’s Integrated General Hospital program, whose stated aim is sustainable acute-to-community care with shared care between hospital and community settings^21^. MIC@Home was first piloted by MOHT in 2019 at two tertiary hospitals, expanded as a regulated “sandbox” from 2022, mainstreamed across Singapore’s public acute hospital system from April 1, 2024, and is now operated by all three regional clusters under cluster-specific brand variants^6,22^.

To put MIC@Home’s scale in context: Singapore’s public acute hospital system comprises nine acute general hospitals (within an 11-hospital public system that also includes a women’s and children’s hospital and a psychiatric hospital), with approximately 9956 public-sector beds out of 12,025 total acute beds nationally as of 2023, equivalent to about 2.0 acute beds per 1000 population^23,24^. MOH’s MIC@Home virtual-bed target of approximately 300 beds by end-2024 represents roughly 3% of the public acute bed base^25^. Notably, MIC@Home is being added on top of, not in place of, a planned expansion of approximately 4000 conventional public acute and community hospital beds by 2030, including the Woodlands Health campus and the new Eastern General Hospital scheduled to open by 2030^25^. MIC@Home patients are eligible for the same subsidies, MediShield Life coverage, MediSave, and Integrated Shield Plan benefits as traditional inpatients^7^. As of end-2023, more than 2000 patients had participated in the program (covering both the 2019 pilot and the 2022 sandbox phase), translating to approximately 9000 hospital bed-days saved^7,25^. The scale asymmetry with the United States also deserves emphasis. Singapore targets roughly 300 virtual beds and had treated about 2000 cumulative patients through end-2023; the United States counts hundreds of approved programs and far larger volumes. Our argument is therefore not that Singapore leads on adoption—the United States plainly does—but that Singapore’s design leads on integration. The exemplar is smaller and younger than the system it is meant to instruct; what it offers is a coherent architecture, not proof at scale.

The program operates through two specific referral pathways: Early Supported Discharge (ESD) for patients transitioning from hospital wards, and Admission Avoidance (AA) for patients who can be admitted directly from emergency departments, outpatient clinics, or primary care. Eligible cases include certain lower- to moderate-acuity medical conditions that can be safely managed at home with a combination of home visits, remote monitoring, and teleconsultation. Care is delivered by multidisciplinary teams including doctors, advanced practice nurses, pharmacists, therapists, and medical social workers^6^.

Peer-reviewed evidence from Singapore’s hospital-at-home pilots is accumulating. Single-arm data from the NUHS@Home pilot established feasibility but could not compare outcomes against ward-based care^26^. A subsequent prospective quasi-experimental evaluation of NUHS@Home (302 patients, 2021–2023) found 42% lower cost per bed day and 24% lower cost per episode than ward-based care, with no significant differences in 30-day readmission, emergency department reattendance, or patient-reported quality of life—though the study was non-randomized, drawn from a single academic health system, and not powered for non-inferiority^27^. MOH has stated that care outcomes under MIC@Home are comparable to hospital-based care, and early qualitative work suggests high patient and caregiver acceptance^7,28^. No independent evaluation of the mainstreamed, system-wide program operating since April 2024 has yet been published; the comparative evidence base derives from pilot-era cohorts.

### D. The Care Continuum: How Healthier SG and MIC@Home Connect

Healthier SG addresses the upstream end of the continuum: keeping patients healthier, managing chronic conditions proactively, and reducing avoidable deterioration. MIC@Home addresses the acute end by delivering selected episodes of hospital-level care in patients’ homes when admission is clinically required. What makes Singapore’s approach instructive for creating a broader care at home continuum is the explicit policy and operational architecture across institutional oversight, digital infrastructure, workforce model, clinical protocols, and financing intended to connect these programs that exist at the two poles (Table 1).

**Table 1:** Shared Foundations Linking Chronic Disease Management (Healthier SG) and Acute Care at Home (MIC@Home)

Dimension	Shared Foundation	How It Connects the Two Programs
**Institutional origin & oversight**	“Three Beyonds” policy framework (2016) and MOHT (est. 2018)	MOHT’s Future Primary Care program built the telehealth/self-management infrastructure underlying Healthier SG; its Integrated General Hospital program designed and scaled MIC@Home. Both are delivered through the same three Regional Health Manager clusters (2017 reorganization), each accountable end-to-end for primary, community, acute, and home-based care in its geography.
**Digital infrastructure**	National Electronic Health Record (NEHR), maintained by Synapxe	NEHR carries admission/visit history, discharge summaries, medication history, and Healthier SG data—a longitudinal backbone for cross-setting coordination (though a fully closed-loop MIC@Home-to-Healthier SG workflow is not yet publicly documented). HealthHub gives patients a parallel interface for records, reminders, and self-management.
**Workforce model**	Teams designed to span settings rather than sit within one	Regional clusters deploy “Healthier SG Teams” (community nurses, wellbeing coordinators) spanning GP clinics, community partners, and hospitals; PCN nurses/care coordinators support private GPs, including during transitions to/from acute care. MIC@Home uses multidisciplinary teams (physicians, APNs, pharmacists, therapists, medical social workers) to deliver hospital-level care at home. MOH’s stated aspiration: post-discharge care “handed over” to the patient’s family physician, some now trained as family medicine specialists for complex chronic disease.
**Clinical protocols**	Jointly developed Healthier SG Care Protocols (12 at launch in 2023; 20 as of 2026)	Developed by MOH with the College of Family Physicians Singapore, Singapore Medical Association, Primary Care Networks, and the three regional clusters; cover chronic conditions (diabetes, hypertension, lipid disorders, etc.) with harmonized referral/data flows. Include forward-referral (primary care → specialist outpatient) and back-referral (hospital specialist → primary care) pathways, supported by Virtual Curbside Consults for direct GP–specialist triage.
**Financing**	Aligned incentives across settings, no penalty for home-based care	Healthier SG: capitated annual service fee, MediSave eligibility for outpatient chronic care, subsidized medications at private clinics—aligning payment with longitudinal accountability. MIC@Home: patients retain the same MediShield Life, MediSave, and Integrated Shield Plan benefits as conventional inpatients. Net effect: neither program financially incentivizes fragmenting care across settings.

Nevertheless, integration at the patient-level handoff remains a work in progress. Public sources do not yet document a routine, protocolized post-discharge transition from MIC@Home to a Healthier SG-enrolled family physician, with structured timelines, named care-transition staff, and shared dashboards tying the two programs together. MOHT’s own programmatic documentation distinguishes MIC@Home (acute substitute), transitional hospital-to-home services, and primary-care home medical services as separate streams, indicating a typology that is not yet fully unified at the operational level^6^. While the strategic framework and structural enablers are in place, a closed-loop patient journey across the continuum has yet to be realized.

### E. Centralized Governance and Its Limits

What makes Singapore’s design and governance “centralized” is not a single mandate but a four-tier stack of aligned authorities. At the apex, the Ministry of Health sets policy, financing rules, regulatory frameworks, and subsidy schemes. MOHT serves as MOH’s dedicated innovation office, designing and scaling cross-setting care models including both Future Primary Care (which underpins Healthier SG) and the Integrated General Hospital program (which houses MIC@Home)^21,29^. The Agency for Integrated Care (AIC) coordinates community care services and administers community-based subsidies that bridge hospital discharge and longitudinal community care. The three Regional Health Manager clusters operate the actual delivery system end-to-end within their geographies. Underlying all four tiers, Synapxe operates the NEHR and the digital platforms that connect them.

This stack enables coherence and scale but introduces tensions. Physician engagement is uneven: while many GPs support Healthier SG’s objectives, some report that documentation, care protocols, and coordination requirements impose significant administrative burden^20^. For MIC@Home, scaling beyond current capacity requires addressing workforce constraints, standardizing clinical protocols across hospital clusters, and integrating third-party service providers^30^. Centralization also risks rigidity: standardized protocols may not accommodate local variation, and top-down mandates can crowd out professional judgment. Fiscal sustainability is another concern: expanded subsidies for Healthier SG and mainstream financing for MIC@Home increase immediate public expenditure, and long-term costs depend on whether preventive care investments reduce downstream hospitalization.

## The U.S. Context: Hospital at Home as a Targeted Solution to Increase Hospital Capacity

### A. The growth and limits of hospital at home

HaH programs in the United States deliver hospital-level acute care (physician oversight, nursing visits, diagnostic testing, intravenous therapies) in patients’ homes. The concept dates back more than two decades, with early programs at academic medical centers demonstrating clinical outcomes comparable to inpatient care, but at lower cost^31,32^. Despite promising evidence, HaH remained a niche model until the COVID-19 pandemic.

In November 2020, CMS launched the Acute Hospital Care at Home (AHCaH) waiver, allowing Medicare-certified hospitals to admit eligible patients to HaH programs and receive reimbursement equivalent to that provided for traditional inpatient stays^3,4^. The response was rapid: as of March 2026, following the waiver’s five-year extension, 366 programs across 139 health systems and 37 states held CMS approval to operate AHCaH programs^33,34^.

Evaluations report similar or lower rates of mortality, readmission, and hospital-acquired complications, alongside high patient satisfaction^35,36^.

While there is national-level policy support for HaH expansion in the U.S., unlike in Singapore, HaH is treated more as a targeted tool for alleviating hospital capacity constraints than as an integrated component of a broader care-at-home continuum. The AHCaH waiver preserved inpatient reimbursement for home-based care, which made programs financially viable but also reinforced the hospital-centric orientation of U.S. healthcare delivery. By conditioning participation on operational requirements designed to mirror an inpatient unit—such as twice-daily in-person visits by registered nurses or mobile integrated health paramedics, twice-daily vital-sign monitoring, rapid emergency response guarantees, and access to diagnostic services including radiology—the waiver ties programs to high fixed operational costs and a narrow acute focus^4^. Consequently, HaH functions predominantly as a mechanism to deploy high-acuity “virtual beds” rather than as an adaptable bridge that connects seamlessly to upstream chronic disease management or downstream longitudinal care^37^.

The federal waiver’s regulatory status has also been uncertain. Originally tied to the COVID-19 public health emergency, AHCaH has been sustained by a series of short-term congressional extensions, with a lapse during the federal government shutdown in 2025, until the Consolidated Appropriations Act, 2026 (Pub. L. No. 119-75, § 6210), signed into law in February 2026, extended the waiver through September 30, 2030^34^. This pattern of reactive extensions underscores a fundamental policy gap: U.S. HaH policy and design remain anchored to a pandemic-era solution to acute hospital bed shortages, sustained through incremental legislative extensions rather than framed as part of a national strategy for leveraging the home as a site for a broader continuum of care transformation.

Moreover, the waiver’s federal authorization does not guarantee state-level implementation: several states, including California, still lack licensing statutes that explicitly authorize acute hospital at home as an inpatient care model, leaving CMS-approved programs unable to operate or forced into lower-acuity alternatives^38–40^. This gap between federal reimbursement authority and state regulatory recognition compounds the waiver’s inherent fragility in supporting a care at home strategy at scale for U.S. health systems.

### B. Emerging components of a care at home continuum

The U.S. has many of the components of an integrated care at home continuum in the form of individual policy mechanisms and health system innovations, although, unlike in Singapore, there is no comparable national framework driving the design of a continuum. HaH programs address the acute care episode but typically do not extend more longitudinally to facilitate the more comprehensive transitions of care that are important for preventing hospital readmissions, improving quality, and reducing total cost of care.

The United States does possess mechanisms, including reimbursement models and national- and state-level care model innovations, that could support a more longitudinal care at home continuum, such as Medicare’s transitional care management and chronic care management billing codes, accountable care organizations including ACO REACH, the GUIDE dementia care model, and PACE^41–45^. Although these mechanisms have been effective in solving for specific care gaps, they have not yet been systematically leveraged at scale by U.S. health systems to build care continuums that leverage the home setting.

Several structural factors contribute to this gap. Payment remains fragmented across fee-for-service, Medicare Advantage, and Medicaid, with limited incentives for integrated longitudinal care. Regulatory authority is split across federal and state levels. Primary care infrastructure is underdeveloped relative to hospital capacity. Digital interoperability, essential for coordinating care across settings, remains a persistent challenge^46^.

Yet the components of a true care-at-home continuum are beginning to emerge. Individual health systems are assembling programs that span chronic care management, remote monitoring, acute care at home, and post-acute transitional care. Many of these innovations notably emerged from health systems in states such as California that have not recognized the federal CMS AHCaH waiver, and that thus had to adapt to build care at home programs that do not rely on the waiver. California health systems such as Kaiser Permanente, Stanford Health Care, Cedars-Sinai, and University of California San Diego Health have launched “non-waiver” care at home programs that enroll lower-acuity populations toward the end of the hospital stay rather than substituting for the entire inpatient admission. This approach has enabled more flexible adaptations than the CMS waiver would have permitted, allowing many of these programs to scale more quickly, extend into management and care coordination further downstream of the hospital stay, and integrate with existing ambulatory programs and home health agencies, all while freeing inpatient hospital beds^47^. Health systems in other states, even those that have the CMS HaH waiver in place, are similarly investing in lower-acuity “hospital to home” care at home programs, in many cases alongside CMS waiver HaH programs, to build a broader care at home continuum that extends beyond just substituting for the acute hospital stay—to reduce hospital readmissions and improve population health metrics, while also continuing to reduce hospital length of stay and increase hospital capacity for these organizations. The emergence of these programs in the U.S. is not a direct result of any federal- or state-level policy mandate, but rather is organically driven by individual health systems responding to organizational needs. Peer dissemination of best practices is also often facilitated through non-governmental networks such as the Hospital at Home Users Group, a national collaborative of health systems and hospital-at-home programs^48^, and interest groups within professional societies such as the Society of Hospital Medicine^49^.

While these programs vary in scope and design, they represent a growing recognition across health systems in the U.S. that the future of care at home is not limited to just AHCaH waiver programs, but can be built as a broader, integrated care continuum.

## Insights for U.S. Health Systems Building a Care at Home Continuum

Singapore’s experience offers several insights for U.S. health systems looking to integrate HaH programs into a broader care at home continuum.

### A. Design programs with a broader care at home strategy

Singapore approaches acute care at home as a component embedded within a broader continuum of chronic disease management and prevention with shared infrastructure and integrated workflows. U.S. health systems that launch HaH programs in isolation risk optimizing one segment of care while leaving the systemic drivers of hospitalization unchanged. Singapore’s policy design suggests that the strategic question is not “How do we build a hospital at home program?” but “How do we construct a care at home model that reduces reliance on hospital-based care across the full trajectory of disease?”

U.S. health systems may explore integrating HaH programs into a broader strategy for transitions of care, population health, and chronic care management that leverages the home as the care setting enabled by digital infrastructure and virtual care. The value drivers for HaH programs, thus, may be expanded beyond just hospital capacity creation to being a component of a broader care continuum that improves quality of care, improves population health metrics, and reduces hospital readmissions. Operationally, this may require more intentionally designed systems that transition patients into and out of HaH programs as part of a broader care at home umbrella that leverages shared operational and digital infrastructure (e.g., remote patient monitoring platforms, virtual nursing) and aligned strategy, with HaH serving as the solution for higher-acuity care at home.

### B. Align payment to incentivize high quality care across the continuum, rather than just substituting hospital care

The AHCaH waiver, by preserving inpatient reimbursement, made HaH financially viable in the U.S. but also preserved a hospital-centric payment logic that does not support care beyond the hospitalization that may otherwise improve quality and reduce cost^36,37^.

Broadening care at home reimbursement strategies beyond the AHCaH waiver to value-based arrangements, such as through accountable care organizations and payer contracts that bundle payments for care episodes, may enable health systems to build and align more flexible care at home models with value drivers. One such approach is the CMS Transforming Episode Accountability Model (TEAM), a payment model launched in 2026 for select acute care hospitals that bundles payments for five surgical episodes—lower extremity joint replacement, surgical hip/femur fracture treatment, spinal fusion, coronary artery bypass graft, and major bowel procedure—spanning the inpatient or outpatient procedure itself plus the 30 days following discharge^50^. TEAM ties a single target price and quality adjustment to the entire episode, creating a direct financial incentive for hospitals to manage recovery, prevent complications, and avoid unnecessary post-acute utilization across the full 30-day window through a comprehensive care at home program that reduces inpatient bed utilization and readmissions. This structure rewards health systems that can extend monitoring, coordination, and rapid response into the home during recovery—capabilities that hospital-at-home infrastructure is well positioned to provide—suggesting that episode-based payment models like TEAM could serve as a more durable, non-waiver-dependent financing pathway for care-at-home models than the AHCaH mechanism currently allows.

### C. Invest in shared digital infrastructure for the care at home continuum

Singapore’s National Electronic Health Record (NEHR) and HealthHub platform reflect a strategic commitment to shared digital infrastructure that connects primary care, hospital care, and home-based services under a single longitudinal record spanning the full continuum. While medical records can be shared between health systems to an extent in the U.S. through health information exchanges (HIEs), electronic health record (EHR) and care management systems are typically designed for a single site of care—i.e., inpatient versus ambulatory—with few modules purpose-built for the transitional period when patients move between hospital, home, and primary care. This leaves a structural gap where a care-at-home continuum most needs coordination: the step-down from acute to home-based care and the handoff back into longitudinal management. Health systems can consider integrating capabilities across the care at home continuum, such as shared remote patient monitoring services, virtual provider and virtual nursing platforms, data analytics infrastructure for risk stratification, and emerging AI capabilities to orchestrate care across settings, treating these as a connected infrastructure layer rather than separate point solutions procured program by program.

### D. Redesign the workforce for home and community settings

Both Healthier SG and MIC@Home contemplate workforce models that differ from traditional hospital staffing. Singapore has expanded the roles of community nurses, care coordinators, allied health professionals, and community volunteers to deliver care at scale outside hospitals^5,51^.

U.S. health systems face acute workforce constraints, particularly in nursing and paramedicine. The transferable mechanism is the boundary-spanning role: Singapore’s Healthier SG Teams and Primary Care Network nurses are employed to work across GP clinics, community partners, and hospitals rather than within a single cost center. The testable U.S. counterpart is to create home-based care roles with explicit dual accountability to both the HaH service and ambulatory or population health functions, and to track retention and cross-setting coverage for those roles as program metrics rather than redeploying hospital staff episodically. It also suggests that non-traditional roles—community health workers, health coaches, trained volunteers—can meaningfully extend system capacity for preventive and chronic care^46,52^.

Equity considerations sharpen this workforce challenge: Singapore’s model rests in part on a social substrate the U.S. largely lacks, including widespread live-in domestic help that extends household caregiving capacity, and the NUHS@Home evaluation’s finding of higher domestic-helper prevalence among home-care patients suggests this support shaped who could be safely cared for at home^27^. Absent that substrate, U.S. workforce redesign must substitute paid, professional caregiving capacity—not assume unpaid family labor—or risk a care-at-home continuum that tracks household resources rather than clinical need.

### E. Align governance and outcomes across legacy silos to span the care continuum

Singapore’s centralized governance enables rapid scaling and coherence, but U.S. health systems must achieve analogous coordination through organizational governance rather than state or federal authority. This means establishing cross-functional leadership that spans HaH, primary care, chronic disease management, and post-acute care; shared metrics that track outcomes across the continuum rather than within individual programs; and feedback mechanisms that allow frontline innovation while maintaining system-wide coherence^53^.

Comparative health systems research suggests that hybrid governance models—combining strategic central guidance with operational autonomy—are most resilient^54,55^. Singapore’s specific mechanism is the four-tier stack described above: policy, innovation, coordination, and delivery vested in distinct but aligned authorities. U.S. health systems may consider investing in aligned leadership and accountability for the care at home continuum, with HaH, chronic care management, and post-acute services reporting shared metrics (escalation rates, 30-day readmission, and time to first primary care contact after discharge) to one governance forum, while local teams retain authority to adapt programs to their populations and markets.

### Limitations and future research directions

This analysis has several limitations. First, much of the evidence on MIC@Home and Healthier SG is early-stage and observational, with limited long-term outcome data. The integration between these programs, the care model we describe, is an emerging policy reality, not yet a fully realized operational system. Second, cross-national lessons are inherently constrained by differences in political institutions, financing structures, scale, and social context. Singapore’s centralized governance, small geography, and high institutional trust create conditions that cannot be directly replicated in the United States. Third, although Healthier SG and MIC@Home sit within a common reform agenda, the evidence base does not yet establish a fully mature, end-to-end model functioning uniformly across settings. Fourth, this analysis focuses on system design and organizational strategy rather than patient-reported outcomes or equity impacts. Fifth, this analysis reflects the authors’ positionality: one author leads a U.S. academic health system’s care-at-home program, and the other advises healthcare organizations; our assessment of U.S. implementation gaps and opportunities is informed, and potentially shaped, by that vantage point. Future research should examine how care-at-home models affect access, disparities, and long-term disease trajectories across socioeconomic groups. Additionally, as U.S. health systems build care-at-home programs, comparative studies of different organizational approaches—and their outcomes—will be essential for identifying best practices.

## Conclusion

Rising chronic disease burdens have exposed the limitations of hospital-centric health systems. Singapore’s response, which aligns primary-care reform and acute care at home within a broader policy agenda, rests on the premise that care at home is most effective when embedded within a wider strategy encompassing prevention, chronic disease management, and community-based support. What Singapore demonstrates to date is design rather than performance: an architectural intent backed by structural enablers, alongside the real challenges of implementation—from provider burden to fiscal sustainability to an integration loop that has yet to fully close.

For U.S. health systems, Singapore’s experience may provide insights that inform efforts to expand HaH programs into a more integrated care at home continuum as the institutional architecture for a fundamentally different model of care delivery.

## Data Availability

No datasets were generated or analysed during the current study.
